# Inflammation characteristics and anti-inflammation treatment with tocilizumab of severe/critical COVID-19 patients: A retrospective cohort study

**DOI:** 10.7150/ijbs.56952

**Published:** 2021-05-17

**Authors:** Qin Hu, Yu Xu, Ying Xiang, Bin Wang, Zhiquan Yuan, Yifan Shan, Wenjing Zhang, Na Wu, Tingting Xia, Chengying Li, Weijia Xie, Xiaoyue Jia, Zubin Yu, Li Bai, Yafei Li

**Affiliations:** 1 Department of Epidemiology, College of Preventive Medicine, Army Medical University (Third Military Medical University), Chongqing 400038, People's Republic of China.; 2 Department of Respiratory and Critical Care Medicine, the Second Affiliated Hospital of Army Medical University, Chongqing 400037, People's Republic of China.; 3 Department of Thoracic Surgery, the Second Affiliated Hospital of Army Medical University, Chongqing 400037, People's Republic of China.

**Keywords:** COVID-19, Severe and critical patients, Inflammatory factors, Anti-inflammation treatment, Tocilizumab

## Abstract

The efficacy of tocilizumab on the prognosis of severe/critical COVID-19 patients is still controversial so far. We aimed to delineate the inflammation characteristics of severe/critical COVID-19 patients and determine the impact of tocilizumab on hospital mortality. Here, we performed a retrospective cohort study which enrolled 727 severe or critical inpatients (≥18 years old) with laboratory-confirmed COVID-19 from Huoshenshan Hospital (Wuhan, China), among which 50 patients received tocilizumab. This study confirmed that most recovered patients manifested relatively normal inflammation levels at admission, whereas most of the deceased cases presented visibly severe inflammation at admission and even progressed into extremely aggravated inflammation before their deaths, proved by some extremely high concentrations of interleukin-6, procalcitonin, C-reactive protein and neutrophil count. Moreover, based on the Cox proportional-hazards models before or after propensity score matching, we demonstrated that tocilizumab treatment could lessen mortality by gradually alleviating excessive inflammation and meanwhile continuously enhancing the levels of lymphocytes within 14 days for severe/critical COVID-19 patients, indicating potential effectiveness for treating COVID-19.

## Introduction

The coronavirus disease 2019 (COVID-19) caused by severe acute respiratory syndrome coronavirus 2 (SARS-CoV-2) infection has been declared by the world health organization (WHO) as a public health emergency of international concern. Apparently, COVID-19 pandemic has profoundly influenced the health of millions of people worldwide with an average case fatality rate of 2.09% by May 3, 2021, from WHO. However, up to present, no effective treatment has been established.

Accumulating evidence strongly suggests that most of the severe COVID-19 patients may manifest the excessive and uncontrolled release of pro-inflammatory cytokines (including interleukin-6 (IL-6), tumour necrosis factor-α, C-reactive protein (CRP)), signifying cytokine storm which could lead to acute respiratory distress syndrome (ARDS) and death 1, 2. Several other studies approved the significant elevation of IL-6, procalcitonin (PCT) and CRP in severe or critical COVID-19 patients 3-5; moreover, a retrospective, multicentre cohort study also observed a notable elevation of serum IL-6 level in deceased COVID-19 patients compared with that of the survivors 6.

Therefore, timely and effective anti-inflammation therapy will have a vital significance to prevent further injury and reduce mortality. At the beginning of the outbreak, however, there's a lack of specific intervention, and the treatment of COVID-19 relies on relieving symptoms and organ support. Tocilizumab, a recombinant anti-human IL-6 receptor (IL-6R) monoclonal antibody, can block IL-6 signaling transduction and its mediated inflammatory response 7, 8, and thus has attracted much attention on its therapeutic effects in COVID-19 patients 9. Several studies from China and Europe have revealed that treatment with tocilizumab could improve clinical outcomes in patients with severe COVID-19 5, 10, 11; meanwhile, Eimer J et al. indicated that treatment with tocilizumab in critical COVID-19 patients might reduce the time of mechanical ventilation and the length of stay in an intensive care unit (ICU) and hospital 12. However, another early study observed that tocilizumab administration did not reduce ICU admission or case fatality rate in a cohort of 21 patients 13. Moreover, a recent study focusing on moderate or severe patients showed that tocilizumab might have lessened the risk of death by day 14, whereas could not find obvious difference on day 28 mortality 14; meanwhile, another randomized trial which only included moderately ill hospitalized patients with COVID-19 also suggested that tocilizumab could not effectively prevent intubation or death 15. Based on these previous studies, we noted that the clinical stages of the disease may impact the efficacy of tocilizumab treatment on the prognosis of COVID-19 patients. More importantly, a systematic review and meta-analysis on critically ill COVID-19 patients 16 showed tocilizumab to be efficacious in reducing mortality. Thus, the efficacy of tocilizumab on the prognosis of severe or critical COVID-19 patients remains controversial and needs to be clarified. Meanwhile, most published and preprint reports lacked persistent attention on specific effects of tocilizumab on inflammatory indicators and immunological factors.

In this cohort study, we firstly did a comprehensive assessment of the inflammation levels in severe or critical COVID-19 patients. We then focused on observation of the effectiveness of tocilizumab in treating severe/critical COVID-19 patients and further explored the specific roles of tocilizumab treatment in inflammatory response and immune function.

## Materials and Methods

### Study design and participants

For this retrospective cohort study, we reviewed the medical records of all adult patients (≥18 years old) who were diagnosed with severely ill or critically ill COVID-19 hospitalized at a specialized hospital, Huoshenshan Hospital, Wuhan, China, between February 3, 2020, and April 14, 2020. All severe/critical inpatients with COVID-19 pneumonia were diagnosed and classified at admission according to “Diagnosis and Treatment Protocol for Novel Coronavirus Infection-Induced Pneumonia (Version seven)” published by the National Health Commission of China 17. Adult severe cases were defined as meeting any of the following three criteria: (1) respiratory distress, respiratory rate (RR) ≥ 30 times/min; (2) oxygen saturation ≤ 93% at resting state; (3) arterial partial pressure of oxygen (PaO2) / oxygen concentration (FiO2) ≤ 300 mmHg. Critical cases were defined as exhibiting any of the following criteria: (1) respiratory failure and requiring mechanical ventilation; (2) shock; (3) with other organ failure and requiring ICU care. Criteria for being discharged were defined as meeting all of the following conditions: (1) body temperature returned to normal for at least three consecutive days; (2) respiratory symptoms eased obviously; (3) pulmonary imaging showed marked absorption of inflammation; (4) nucleic acid test was negative for two consecutive times on respiratory tract samples, and the sampling interval was not less than 24 hours. This study protocol was reviewed and approved by the Ethics Review Committee of Huoshenshan Hospital, which waived the requirement for informed consent.

### Data collection

All data were obtained from reviewing the electronic medical record system. Using a standardized uniform form, we extracted demographic and clinical characteristics, dates of hospital admission and discharge/death, clinical outcomes etc. Treatment information included antiviral therapies, antibiotics, use of tocilizumab, corticosteroids, convalescent plasma, traditional Chinese medicine and other supporting treatments; specially, we collected the date and dosage of tocilizumab treatment in detail. Laboratory data included blood routine examination, IL-6, CRP and PCT, hypersensitive C-reactive protein (hs-CRP) and so on. We recorded these laboratory indices twice, including the first-test values within 24 h after admission and the last-test values before outcomes, and subsequently calculated the differences between them; moreover, we repeatedly recorded the values of laboratory indices at a few time points after tocilizumab treatment in propensity score-matched cohort. Two physicians (Xu and Wang) abstracted and checked all data.

### Statistical analysis

Continuous variables were presented as median (interquartile range [IQR]) and compared for statistical difference with a Mann-Whitney U test; categorical variables were presented as number (%) and compared by a *χ*² test or Fisher's exact test. To perform Cox proportional hazards regression analysis, some quantitative variables were transformed into categorical variables according to their reference ranges. A univariable Cox regression analysis was used to screen potential confounders, and then the significant variables were retained as covariates in subsequent analyses. A multivariable Cox regression model was used to assess the association between tocilizumab treatment and risk of death.

To minimize the effects of confounding factors (age, gender, pre-existing comorbidities, baseline disease severity at admission and other treatments), a propensity score 1:1 matching method was performed to match patients using tocilizumab with patients not using any form of tocilizumab within a matching calliper of 0.05 standard deviation (Characteristics of the cohort before and after matching were presented in **Table S3**). The statistical difference between the tocilizumab group and the no tocilizumab group in the propensity score matching (PSM) cohort was compared using the McNemar test for categorical variables and Wilcoxon signed ranks test for continuous variables. Survival curves were drawn using the corresponding Cox model accounting for tocilizumab as a time-varying exposure and with adjustment for confounding bias.

All tests were 2-sided, and a P<0.05 was considered to be statistically significant*.* SPSS statistical software (version 26.0, IBM) was used for statistical analyses and Prism Software (GraphPad 8.0) for drawing figures.

## Results

### Baseline characteristics of severe and critical inpatients with COVID-19

A total of 744 severely or critically ill patients with confirmed COVID-19 were included in this study. As of 14 Apr 2020, 42 of these patients had died of COVID-19 during hospitalization and 685 patients recovered and discharged; 17 patients were lost to follow-up and excluded. Thus, we included 727 severe/critical patients with COVID-19 in the final analysis. As shown in **Table 1**, Overall 53.8% were male and the median age was 65.0 years (range, 56.0 to 72.0). Of these, nearly two-thirds of 727 patients presented at least one comorbidity among which hypertension (43.9%) was the most common, followed by diabetes (20.1%), other diseases (14.4%) and coronary heart disease (11.0%). The median time from illness onset to this hospital admission or to the outcomes (death or discharge) was 28.0 (15.0-40.0) days and 45.0 (34.0-57.0) days, respectively. For all discharged patients, the median time from hospital admission to discharge was 15.0 (9.0-22.0) days.

Among 727 patients with severe/critical COVID-19, 50 (6.87%) patients received tocilizumab treatment and one of them died; all 50 patients in the tocilizumab group received intravenous tocilizumab at least once during hospitalization; among them, one received a total of 800 mg tocilizumab treatment, 34 received 400 mg, 4 received 320 mg, 3 received 240 mg, and 8 received 80 mg. Numerically fewer deceased patients (2.38%) used tocilizumab compared with the recovered patients (7.15%) (**Table 1**), but the difference was not statistically significant. The majority of all 727 patients used traditional Chinese medicine (n=629, 86.5%) and antiviral drugs (n=422, 58.0%) that included oseltamivir, lopinavir/ritonavir and arbidol. More than a third (n=315, 43.3%) of 727 patients received antibiotic treatment. Nearly a quarter of patients used corticosteroid, including methylprednisolone, hydrocortisone and dexamethasone. Convalescent plasma was given to 81 (11.1%) patients, and blood purification therapy was given to 6 (0.8%) patients (**Table 1**).

### Inflammation-related laboratory parameters

**Figure 1:**
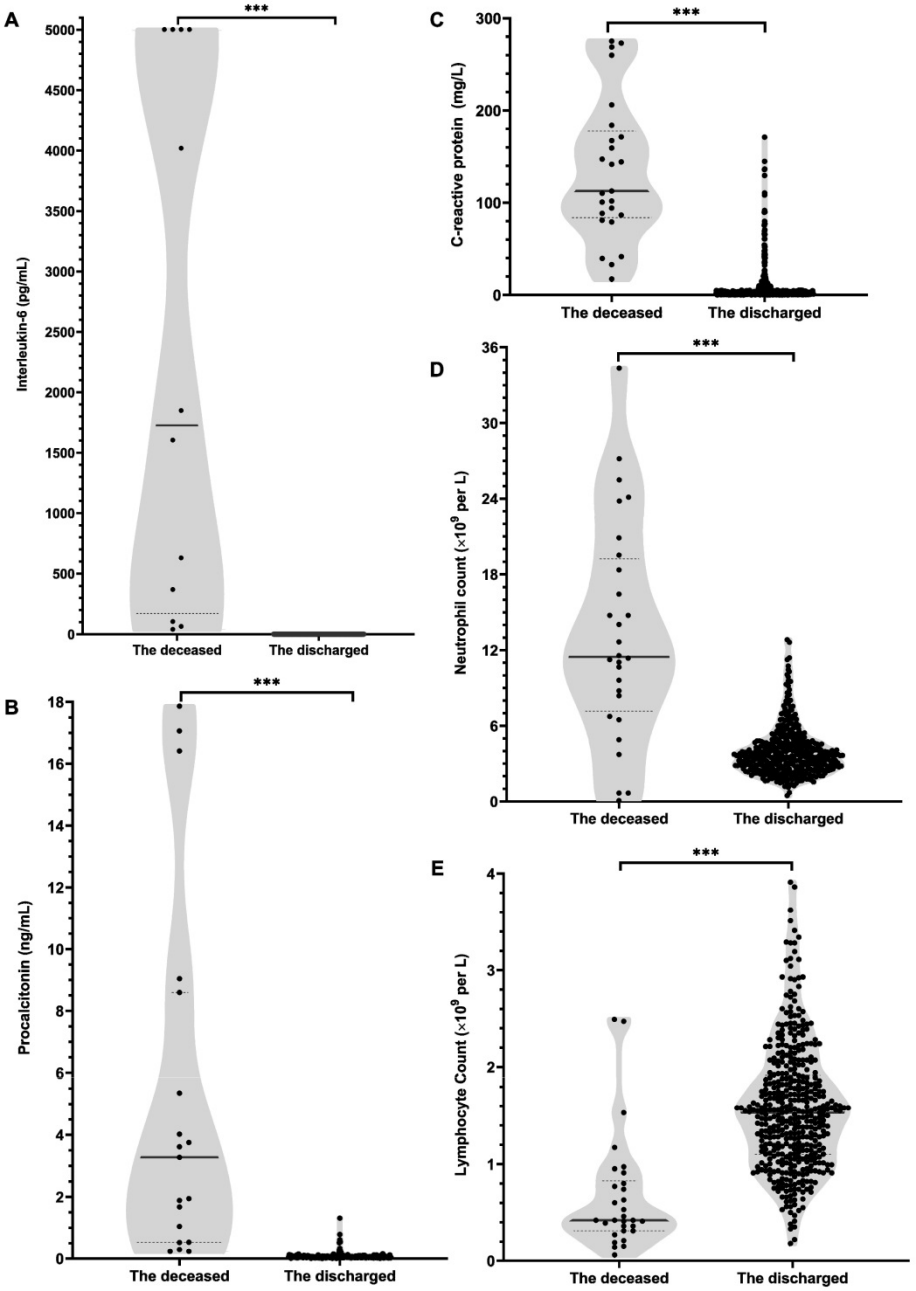
** Levels of inflammation-related laboratory indicators measured before their deaths in deceased patients or before being discharged in recovered patients.** A, interleukin-6; B, procalcitonin; C, C-reactive protein; D, neutrophil count; E, lymphocyte count. Value for each indicator of each patient was shown in black dots. For interleukin-6 (A), the values in some deceased individuals exceeded the upper limit of detection, expressed by the upper limit of 5000. Bar represents the median (IQR) of each group. Differences between the deceased and the discharged patients were compared by using Mann-Whitney U test. ^***^, P<0.001.

According to the first-tested values after hospital admission and the last-tested values before outcomes (**Table S1; Figure 1**), the deceased patients, compared with recovered patients, had sharply lower lymphocytes count and lymphocytes ratio at admission and more severe lymphopenia were observed before their deaths. Meanwhile, the data showed that the monocyte ratio was remarkably lower in deceased patients than recovered patients no matter at admission or before outcomes. On the contrary, white blood cell counts (WBC), neutrophil count and neutrophil ratio were notably higher in deceased patients. Accordingly, the deceased patients had an observably higher neutrophil to lymphocyte ratio (NLR) both at admission and before outcomes. Remarkably, excessively high IL-6 level was observed in deceased patients on admission, which was more than 30 times that of recovered patients, and some deceased cases even exhibited extremely high IL-6 values a few days before they died (**Table S1; Figure 1**). Besides, the concentrations of PCT, CRP and hs-CRP in deceased patients were notably higher than those in recovered patients no matter on admission or before outcomes (**Table S1**). Furthermore, the deceased patients than the recovered patients presented the notably greater difference between the last-tested values and the first-tested values in the concentrations of WBC, neutrophil ratio, neutrophil count, lymphocyte ratio, lymphocyte count, monocyte ratio, NLR, IL-6, PCT, CRP and hs-CRP (**Table 1**).

Actually, the values of laboratory parameters in most recovered patients were almost within the reference ranges no matter at admission and before they were discharged (**Figure 1; Table S1**), whereas the concentrations of IL-6, PCT, CRP and neutrophil count at admission in deceased patients were visibly higher than the reference ranges and even progressed into extremely high values before their deaths (**Figure 1; Table S1**).

### Association between tocilizumab and mortality among severe/critical patients with COVID-19

As shown in **Table S3,** before PSM, compared with the non-tocilizumab group, patients who received tocilizumab were significantly older and more likely to have comorbidities; meanwhile, the proportions of patients in the tocilizumab group receiving corticosteroids, convalescent plasma, antibiotics, intravenous albumin and non-invasive mechanical ventilation therapy were significantly higher than those in the non-tocilizumab group. The crude death rate during hospitalization was 2.00% in tocilizumab users versus 6.06 % in no tocilizumab users. Using a Cox model accounting for tocilizumab as a time-varying exposure and with adjustment for baseline differences (including age, gender, disease severity (severe and critical), comorbidities and other treatments), the hazard ratio for death in tocilizumab group as compared with no tocilizumab group was 0.103 (95% CI, 0.013 to 0.798; P = 0.030) (**Table 3**).

In the PSM cohort, matched for various variables (including age, gender, comorbidities and other treatments), the crude death rate in tocilizumab group (death rate 2.00%) was numerically lower than that in no tocilizumab group (death rate 12.00%), but the difference was not statistically significant (**Table 4**). Using a Cox model with adjustment of age, coronary heart disease, the use of corticosteroids and the use of traditional Chinese medicine, the hazard ratio for death in tocilizumab group as compared with no tocilizumab group was 0.038 (95% CI, 0.002 to 0.588; P = 0.019) (**Table 3; Figure S1**). These results indicate that tocilizumab treatment is associated with a lower risk of mortality among severe/critical patients with COVID-19.

### Effect of tocilizumab on inflammatory response in severe/critical COVID-19 patients

**Figure 2:**
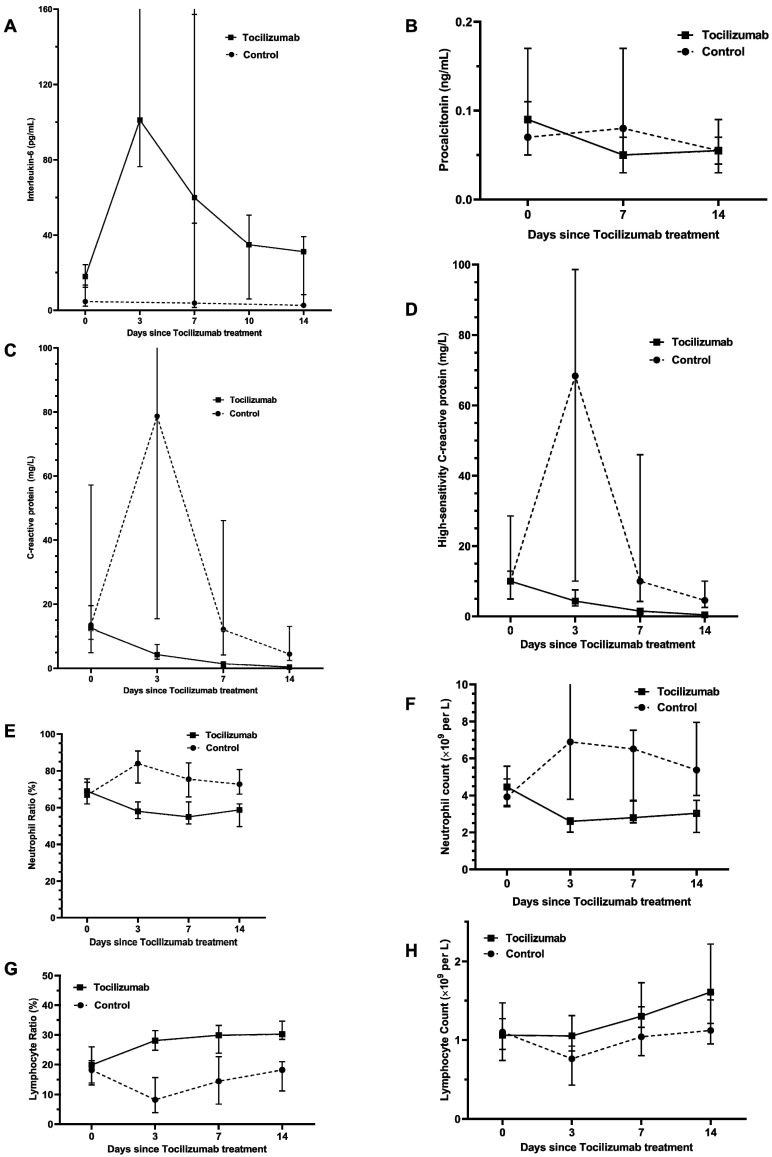
** Effect of tocilizumab treatment within 14 days on various inflammation factors in patients hospitalized with severe/critical COVID-19.** Figure shows temporal changes since tocilizumab treatment in interleukin-6 (A), procalcitonin (B), C-reactive protein (C), high-sensitivity C-reactive protein (D), neutrophil ratio (E) neutrophil count (F), lymphocyte ratio (G) and lymphocyte count (H). Differences between the tocilizumab group versus the non-tocilizumab group were significant for all time points shown, except for day 0 after tocilizumab treatment for procalcitonin, CRP, neutrophil ratio, neutrophil count, lymphocyte ratio and lymphocyte count, and day 14 after tocilizumab treatment for procalcitonin. The statistical difference between the tocilizumab group and the non-tocilizumab group were compared by using Wilcoxon signed ranks test.

Based on PSM analysis in **Table 4**, compared with no tocilizumab group, the tocilizumab group presented a significantly bigger difference between the last-tested values and the first-tested values after admission in the levels of WBC, neutrophil count and neutrophil ratio during hospitalization, whereas presented significantly smaller difference in the levels of lymphocyte ratio. Moreover, as shown in **Figure 2** and **Table S4**, after tocilizumab treatment, the levels of neutrophil ratio, neutrophil count, CRP, hs-CRP and PCT gradually declined and ultimately returned to reference range over time. Strikingly, about within 3 days after using tocilizumab, the concentration of IL-6 temporarily rose, for its receptors blocked by tocilizumab; then, the concentration of IL-6 would progressively decline during days 7 to 14. Furthermore, the level of lymphocyte ratio in the tocilizumab users was notably lower at hospital admission than that in no tocilizumab users, and meanwhile, tocilizumab treatment continuously increased lymphocytes count and lymphocytes ratio within 14 days (**Figure 2; Table S4**). These results indicate that tocilizumab treatment can effectively alleviate inflammation and regulate immune function for severe/critical COVID-19 patients.

## Discussion

Given the fact that inflammatory cytokine storm originating from SARS-CoV-2 invasion is more commonly detected in severe/critical cases with COVID-19 than ordinary cases and often results in disease deterioration and even death 2, 18, 19, analyzing inflammatory characteristics and summing up the experience of anti-inflammation treatments for severe/critical patients is helpful to deepen our understanding of the pathogenic mechanism and contribute to their clinical treatments and disease rehabilitation. The objective of this study was to identify inflammatory features of severe/critical COVID-19 patients and explore the anti-inflammatory effect of tocilizumab therapy on the prognosis of severe/critical patients with COVID-19 during hospitalization. We confirmed that the inflammation in the deceased patients was significantly elevated at admission and kept getting worse during hospitalization, which accelerated their deaths. Moreover, we further observed that tocilizumab treatment was effective for alleviating death in severe/critical patients with COVID-19 across multivariate Cox proportional-hazards before and after PSM. Further exploration reveals that the protective benefits in severe/critical patients might attribute to tocilizumab alleviating inflammation and enhancing immune function, which may shed light on the therapeutic potential for severe/critical COVID-19 patients.

Although there have been accumulating reports describing the inflammation in patients with COVID-19 recently, our study is the first one to analyze in detail the association between clinical outcomes and the baseline levels at admission or the changes of inflammatory levels during hospitalization in severe/critical COVID-19 patients. Circulating CRP, IL-6, PCT and neutrophil counts were inflammation biomarkers that represent the overall status of inflammation 20, 21. The laboratory data in this study, no matter from the first tests or the last tests after admission, strongly confirmed that the levels of inflammation-related indicators (including IL-6, PCT, CRP, hs-CRP, neutrophil ratio and neutrophil count) in deceased patients with severe/critical COVID-19 were remarkably higher than that in recovered patients and positively correlated with the incidence of mortality. More strikingly, the inflammation in deceased patients was steadily aggravated during hospitalization and even presented extremely high concentrations of IL-6, PCT, CRP and neutrophil count that resembles cytokine storm a few days before they died. Similarly, the deceased patients also often presented lymphocytopenia at admission, indicating a tremendous amount of immune cells consumed and the immune function weaken consistently 21, 22, and had undergone a continuous decline until death. Overall, these data, together with previous studies, indicate that excessive and steadily aggravating inflammation or lymphocytopenia will be critical to lead to deleterious clinical manifestations after SARS-CoV-2 infections 2 and monitoring the changes of the inflammation-related indicators can be vital to judging the improvement or deterioration of the disease. On the other hand, timely and effective anti-inflammation intervention may be conducive to arrest the aggravation of the illness and reduce the mortality of severe/critical COVID-19 patients.

Subsequently, this study explored the correlation between anti-inflammation treatments and the risk of death in severe/critical patients with COVID-19. As we all know, IL-6 is one of the crucial proinflammatory cytokines 23, and besides our findings consistent with prior literature supported that the excessive release of IL-6 could be a hallmark and important driving force of cytokine storm which in turn would result in the poor prognosis of COVID-19 patients 6, 24. Tocilizumab, an IL-6 monoclonal antibody, in view of the anti-inflammatory effect, might be an effective treatment in COVID-19 patients 12, 25. Actually, Luo, et al. recommended that the repeated dose of the tocilizumab would be helpful for critically ill patients with elevated IL-6 26. However, a recent study that included moderate and severe patients reported that patients treated with tocilizumab might have a lower risk of noninvasive ventilation, mechanical ventilation, or death by day 14, but did not present a difference on day 28 mortality 14. Kow et al. confirmed that the administration of tocilizumab did not obtain significant mortality benefits although their meta-analysis approved the use of tocilizumab reducing the likelihood of progression to mechanical ventilation and/or all-cause mortality among COVID-19 patients 27; similarly, another randomized trial showed tocilizumab unfruitful in preventing death in moderately ill COVID-19 patients. It is worth noting that most previous studies included not only severe patients but also moderately ill patients, and the randomized trial 15 also only enrolled moderate patients. All these lines of evidence imply that the clinical stages of the disease might impact the effectiveness of tocilizumab treatment in COVID-19 patients. Differently, in our study, both severe and critical patients with COVID-19 were included and all patients had a definite outcome (recovery or death). Our results showed that tocilizumab treatment was associated with a lower risk of mortality, whereas were not effective for shortening time on the length of hospital stay or the time from illness onset to outcomes. In fact, consistent with our study, a meta-analysis 28 which included 38 studies reported that tocilizumab treatment is associated with a reduction of mortality from COVID-19, although tocilizumab treatment did not alter the severity of COVID-19 and the length of hospital stay. Similarly, a systematic review and meta-analysis on critically ill COVID-19 patients 16 showed tocilizumab to be efficacious in reducing mortality; moreover, another systemic review and meta-analysis also confirmed the effectiveness of tocilizumab treatment in reducing the risk of admission to ICU, use of ventilation and mortality 29. Besides, a study in Italy demonstrated that tocilizumab could increase survival and favorable clinical course if used relatively early during COVID-19 with severe respiratory syndrome 30. Thus, in combination with previous studies, we approved that tocilizumab may show potential effectiveness to treat severe/critical COVID-19 patients, and meanwhile the choice of the population (severe or critical) and time window for the use of tocilizumab in the future does matter.

Furthermore, we explored the possible mechanisms of tocilizumab in declining the risk of mortality in severe/critical COVID-19 patients. Considering tocilizumab preventing IL-6 itself from binding to its receptor and alleviating the inflammatory responses 1, we explored the changes of inflammatory hallmarks in tocilizumab users with severe/critical COVID-19. In subjects with PSM matched baseline differences, the dynamic trajectories of IL-6, PCT, CRP, hs-CRP and neutrophil count showed a downward trend and reached the lowest levels within 14 days after tocilizumab treatment. Additionally, tocilizumab users could always present a relatively lower level of inflammatory response after tocilizumab therapy than no tocilizumab users within 14 days. Furthermore, considering other (overlapping) mechanisms which tocilizumab could perform in SARS-CoV-2 infection: immune function regulation, we investigated the effects of tocilizumab on immunological factors and found that using tocilizumab enhanced immune function of COVID-19 patients during hospitalization by progressively increasing lymphocytes count and lymphocytes ratio within 14 days. Therefore, this retrospective cohort study pointed out that tocilizumab treatment could significantly mitigate the excessive inflammation of severe/critical COVID-19 patients, which could support the presence of similar mechanisms in treating COVID-19 with tocilizumab, as reported in earlier research 10.

Our results should be cautiously interpreted due to several limitations. First, due to the observational study design, although we performed multivariable Cox regression and PSM analysis to carefully adjust the potential confounders, the unmeasured confounding factors cannot be excluded. Second, the sample sizes of deceased patients and patients who used tocilizumab during hospitalization were both relatively small. Finally, because our data are from real-world clinical data, it is difficult to rule out information bias caused by missing and inaccurate data.

## Conclusions

In summary, this study systematically described the characteristic and progression of the inflammatory response in patients with severe/critical COVID-19, approving that excessive and further aggravating inflammation could trigger disease deterioration and death. We then confirmed that tocilizumab therapy was effective for diminishing death in severe/critical patients with COVID-19; furthermore, repressing excessive inflammation and enhancing immune function would underlie tocilizumab-associated improved prognosis of severe/critical COVID-19. Our preliminary results, which provide an important clue to the choice of the population (severe or critical) and stratified use of tocilizumab in treating COVID-19, need to be validated by well-designed randomized clinical trials.

## Supplementary Material

Supplementary figures and tables.

## Figures and Tables

**Table 1:** Characteristics of severe/critical patients with COVID-19.

Parameters	Total (n=727)	Deaths (n=42)	Recovered patients (n=685)	P value
**Clinical Characteristics**				
**Age, years**	65.0 (56.0-72.0)	75.0 (64.7-81.2)	64.0 (56.0-72.0)	<0.001
**Sex**				0.041
Male	391 (53.8%)	29 (69.0%)	362 (52.8%)	
Female	336 (46.2%)	13 (31.0%)	323 (47.2%)	
**Classification of disease severity at admission**				<0.001
Severe	690 (94.9%)	25 (59.5%)	665 (97.1%)	
Critical	37 (5.1%)	17 (40.5%)	20 (2.9%)	
**Smoker history**	52 (7.2%)	4 (9.5%)	48 (7.0%)	0.539
**Time from illness onset to hospital admission, days**	28.0 (15.0-40.0)	12.0 (9.5-21.5)	29.0 (15.0-40.0)	<0.001
**Time from hospital admission to death or discharge, days**	15.0 (8.0-22.0)	10.0 (6.0-16.2)	15.0 (9.0-22.0)	<0.001
**Time from illness onset to death or discharge, days**	45.0 (34.0-57.0)	26.0 (18.5-38.0)	46.0 (35.0-57.0)	<0.001
**Comorbidity**	469 (64.5%)	38 (90.5%)	431 (62.9%)	0.001
Hypertension	319 (43.9%)	20 (47.6%)	299 (43.6%)	0.615
Coronary heart disease	80 (11.0%)	11 (26.2%)	69 (10.1%)	0.001
Diabetes	146 (20.1%)	14 (33.3%)	132 (19.3%)	0.027
Other cardiovascular diseases	82 (11.3%)	11 (26.2%)	71 (10.4%)	0.002
COPD	13 (1.8%)	6 (14.3%)	7 (1.0%)	<0.001
Other chronic lung illness	35 (4.8%)	4 (9.5%)	31 (4.5%)	0.142
Other diseases	105 (14.4%)	12 (28.6%)	93 (13.6%)	0.007
**Treatments**				
Antibiotics	315 (43.3%)	38 (90.5%)	277 (40.4%)	<0.001
Antivirals	422 (58.0%)	30 (71.4%)	392 (57.2%)	0.070
Intravenous Albumin	133 (18.3%)	31 (73.8%)	102 (14.9%)	<0.001
Convalescent Plasma	81 (11.1%)	14 (33.3%)	67 (9.8%)	<0.001
Blood purification	6 (0.8%)	5 (11.9%)	1 (0.1%)	<0.001
Tocilizumab	50 (6.9%)	1 (2.4%)	49 (7.2%)	0.351
Corticosteroid	185 (25.4%)	33 (75.6%)	152 (22.2%)	<0.001
Traditional Chinese medicine	629 (86.5%)	20 (47.6%)	609 (88.9%)	<0.001
Hemodialysis	5 (0.7%)	4 (9.5%)	1 (0.1%)	<0.001
Loop support	22 (3.0%)	19 (45.2%)	3 (0.4%)	<0.001
Nasal catheter oxygen	577 (79.4%)	35 (83.3%)	542 (79.1%)	0.513
High flow nasal cannula oxygen	78 (10.7%)	30 (71.4%)	48 (7.0%)	<0.001
Non-invasive mechanical ventlilation	54 (7.4%)	31 (73.8%)	23 (3.4%)	<0.001
Invasive mechanical ventlilation	32 (4.4%)	26 (61.9%)	6 (0.9%)	<0.001
**Laboratory findings (reference range)**				
White blood cell count,×10⁹/L (3.5-9.5)				
Changes	-0.10 (-1.70-1.20)	6.55 (-0.95-14.18)	-0.20 (-1.70-1.00)	<0.001
Neutrophil ratio, % (40-75)				
Changes	-4.40 (-14.30-1.40)	3.25 (-0.35-14.90)	-5.50 (-14.90-0.80)	<0.001
Lymphocyte ratio, % (20-50)				
Changes	4.00 (-1.20-11.25)	-2.75 (-5.33-0.25)	5.00 (-0.40-11.60)	<0.001
Monocyte ratio, % (3-10)				
Changes	0.20 (-1.20-2.00)	-1.40 (-4.50-(-0.08))	0.30 (-1.00-2.10)	<0.001
Neutrophil count, ×10⁹/L (1.8-6.3)				
Changes	-0.36 (-1.76-0.67)	6.92 (-0.60-14.34)	-0.41 (-1.77-0.48)	<0.001
Lymphocyte count, ×10⁹/L (1.1-3.2)				
Changes	0.25 (-0.05-0.56)	-0.05 (-0.42-0.18)	0.28 (-0.03-0.61)	<0.001
Monocyte count, ×10⁹/L (0.1-0.6)				
Changes	0.01 (-0.10-0.12)	0.03 (-0.17-0.18)	0.01 (-0.09-0.11)	0.896
Neutrophil/Lymphocyte ratio				
Changes	-0.63 (-2.97-0.14)	3.43 (-6.55-14.56)	-0.68 (-2.94-0.10)	0.017
Interleukin-6, pg/mL (<7)				
Changes	0.40 (-2.86-6.63)	1743.90 (31.55-4471.50)	0.29 (-3.03-4.96)	0.002
Procalcitonin, ng/mL (0-0.05)				
Changes	-0.01 (-0.04-0.01)	2.37 (0.24-8.83)	-0.01 (-0.05-0.00)	<0.001
C-reactive protein, mg/L (0-5)				
Changes	-2.46 (-17.94-0.04)	29.54 (1.98-128.33)	-2.96 (-21.37-(-0.08))	<0.001
hs-CRP, mg/L (0-5)				
Changes (No. <0) ^α^	276/423 (65.25%)	1/21 (4.18%)	275/402 (68.41%)	<0.001

Data are median (IQR), or n (%), or n/N (%). Changes mean the difference between last-tested value and first-tested value after admission. P values were calculated by Mann-Whitney U test, χ² test, or Fisher's exact test, as appropriate. <0 ^α^ means that the value after the last-tested value minus the first-tested value is less than 0. IQR=interquartile range; hs-CRP=High sensitivity C-reactive protein; COPD=chronic obstructive pulmonary disease.

**Table 2:** Inflammatory-related laboratory indicators in deceased patients with severe/critical COVID-19.

Parameters	First test after admission (n=42)	Last test after admission (n=42)	P value
White blood cell count, ×10⁹/L	8.60 (6.60-13.70)	12.30 (7.43-21.38)	0.004
Neutrophil ratio, %	88.20 (78.00-92.50)	93.00 (88.15-95.68)	0.003
Lymphocyte ratio, %	6.30 (4.20-10.80)	3.65 (2.65-7.53)	0.009
Monocyte ratio, %	4.40 (2.10-6.30)	2.05 (1.20-4.18)	<0.001
Neutrophil count, ×10⁹/L	7.53 (5.56-12.48)	11.45 (6.67-19.85)	0.002
Lymphocyte count, ×10⁹/L	0.57 (0.43-1.03)	0.42 (0.31-0.83)	0.480
Monocyte count, ×10⁹/L	0.37 (0.21-0.62)	0.31 (0.13-0.53)	0.579
Neutrophil/Lymphocyte ratio	13.21 (7.88-17.83)	16.05 (8.97-33.16)	0.306
Interleukin-6, pg/mL	83.73 (39.39-185.28)	1848.00 (216.32-5000.00+ ^α^)	0.028
Procalcitonin, ng/mL	0.28 (0.12-0.81)	3.44 (0.66-8.93)	0.001
C-reactive protein, mg/L	72.75 (42.67-138.96)	112.53 (83.59-177.50)	0.003
hs-CRP, mg/L (No. of value >5 mg/L)	31/42 (73.81%)	25/42 (59.52%)	0.040

Data are median (IQR), or n/N (%). P values were calculated by Mann-Whitney U test. + ^α^ means that the values exceeded the upper limit of detection. IQR=interquartile range; hs-CRP=High sensitivity C-reactive protein.

**Table 3:** Hazard ratios for in-hospital mortality in tocilizumab group versus non-tocilizumab group.

	Unmatched		Matched	
	Cox Proportional Hazards Model		Cox Proportional Hazards Model	
	HR^a^ (95% CI)	P value ^a^	HR^b^ (95% CI)	P value ^b^
**Tocilizumab versus**	0.103	0.030	0.038	0.019
**Non-tocilizumab**	(0.013-0.798)		(0.002-0.588)	

^a^HR and P value were calculated using Cox proportional hazards model with adjustment of age, gender, classification of disease severity, comorbiditis, antibiotics, convalescent plasma, corticosteroids, blood purification, hemodialysis and traditional Chinese medicine. ^b^HR and P value were calculated using Cox proportional hazards model with adjustment of age, coronary disease, corticosteroids and traditional Chinese medicine. HR = Hazard Ratio; CI = Confidence Intervals.

**Table 4:** Propensity score matching analysis on tocilizumab treatment group comparing with non-tocilizumab group.

Parameters	Non-tocilizumab (n=50)	Tocilizumab (n=50)	P value^*a*^
**Clinical outcomes**			
Mortality	6 (12.0%)	1 (2.0%)	0.125
Hospital length of stay, days	25.0 (11.7-33.7)	29.0 (19.5-40.0)	0.062
Time from illness onset to outcome, days	48.5 (36.7-59.2)	60.5 (48.7-67.7)	0.001
**Laboratory findings**			
White blood cell count, ×10^9^/L			
First test	6.45 (4.80-8.45)	7.10 (5.05-9.40)	0.242
Changes	0.60 (-1.50-2.75)	-1.50 (-3.08-0.28)	0.002
Neutrophil ratio, %			
First test	68.05 (58.85-85.55)	73.00 (67.50-84.60)	0.059
Changes	-3.60 (-14.75-1.40)	-15.95 (-25.98-(-7.13))	0.002
Lymphocyte ratio, %			
First test	19.55 (8.28-28.83)	15.00 (7.30-21.60)	0.019
Changes	5.10 (-1.30-10.10)	10.70 (5.25-20.05)	0.001
Monocyte ratio, %			
First test	7.70 (4.70-9.45)	7.10 (5.70-9.00)	0.845
Changes	0.10 (-1.88-2.45)	0.95 (-0.73-4.13)	0.123
Neutrophil count, ×10^9^/L			
First test	3.93 (2.97-6.60)	5.36 (3.42-8.25)	0.069
Changes	0.08 (-1.66-2.20)	-1.71 (-3.74-(-0.40))	0.003
Lymphocyte count, ×10^9^/L			
First test	1.10 (0.63-1.55)	0.98 (0.59-1.38)	0.348
Changes	0.43 (0.14-0.90)	0.41 (0.10-0.90)	0.480
Monocyte count, ×10^9^/L			
First test	0.45 (0.34-0.59)	0.50 (0.35-0.66)	0.161
Changes	0.07 (-0.02-0.24)	-0.04 (-0.13-0.12)	0.018
Neutrophil:Lymphocyte ratio			
First test	3.31 (2.05-10.65)	5.21 (3.10-13.28)	0.216
Changes	-0.63 (-6.50-0.18)	-2.32 (-9.42-(-0.71))	0.256
IL-6, pg/mL			
First test	4.49 (2.16-13.45)	21.38 (10.26-58.26)	0.009
Changes	-0.80 (-5.32-0.64)	20.06 (-24.37-64.03)	0.161
Procalcitonin, ng/mL			
First test	0.08 (0.04-0.14)	0.08 (0.05-0.15)	0.351
Changes	-0.01 (-0.05-0.03)	-0.04 (-0.10-(-0.01))	0.157
C-reactive protein, mg/L			
First test	14.53 (1.73-66.63)	28.13 (6.39-87.05)	0.516
Changes	-9.50 (-66.95-(-0.81))	-26.10 (-85.09-(-3.91))	0.525
hs-CRP, mg/L			
First test	10.00+ (1.73-10.00+)	10.00+ (6.39-10.00+ ^b^)	0.031
Changes	-1.93 (-7.08-0.00)	-7.22 (-9.49-(-0.57))	0.112

Data are n (%) or median (IQR). First test means first-tested value after admission and changes mean the difference between last-tested value and first-tested value after admission. ^a^ P values were compared by McNemar test, Wilcoxon signed ranks test or Fisher's exact test, ^b^ + means that the values exceeded the upper limit of detection.

## Abbreviations

ARDS: acute respiratory distress syndrome
CI: confidence interval
COPD: chronic obstructive pulmonary disease
COVID-19: Coronavirus Disease-19
CRP: C-reactive protein
CVD: cardiovascular disease
HR: hazard ratio
hs-CRP: hypersensitive C-reactive protein
ICU: intensive care unit
IL-6: interleukin-6
IMV: invasive mechanical ventilation support
IQR: interquartile range
NLR: neutrophil-to-lymphocyte ratio
PCT: procalcitonin
PSM: propensity score matching
SARS-CoV-2: severe acute respiratory syndrome coronavirus-2
RR: respiratory rate
WBC: white blood cell counts

## References

[1] Zhang W, Zhao Y, Zhang F, Wang Q, Li T, Liu Z (2020). The use of anti-inflammatory drugs in the treatment of people with severe coronavirus disease 2019 (COVID-19): The Perspectives of clinical immunologists from China. Clin Immunol.

[2] Channappanavar R, Perlman S (2017). Pathogenic human coronavirus infections: causes and consequences of cytokine storm and immunopathology. Semin Immunopathol.

[3] Wu C, Chen X, Cai Y, Xia J, Zhou X, Xu S (2020). Risk Factors Associated With Acute Respiratory Distress Syndrome and Death in Patients With Coronavirus Disease 2019 Pneumonia in Wuhan, China. Jama Intern Med.

[4] Chen N, Zhou M, Dong X, Qu J, Gong F, Han Y (2020). Epidemiological and clinical characteristics of 99 cases of 2019 novel coronavirus pneumonia in Wuhan, China: a descriptive study. Lancet.

[5] Xu X, Han M, Li T, Sun W, Wang D, Fu B (2020). Effective treatment of severe COVID-19 patients with tocilizumab. Proc Natl Acad Sci U S A.

[6] Zhou F, Yu T, Du R, Fan G, Liu Y, Liu Z (2020). Clinical course and risk factors for mortality of adult inpatients with COVID-19 in Wuhan, China: a retrospective cohort study. The Lancet.

[7] Wolf J, Rose-John S, Garbers C (2014). Interleukin-6 and its receptors: a highly regulated and dynamic system. Cytokine.

[8] Kaly L, Rosner I (2012). Tocilizumab - a novel therapy for non-organ-specific autoimmune diseases. Best Pract Res Clin Rheumatol.

[9] Zhang C, Wu Z, Li J, Zhao H, Wang G (2020). The cytokine release syndrome (CRS) of severe COVID-19 and Interleukin-6 receptor (IL-6R) antagonist Tocilizumab may be the key to reduce the mortality. Int J Antimicrob Ag.

[10] Toniati P, Piva S, Cattalini M, Garrafa E, Regola F, Castelli F (2020). Tocilizumab for the treatment of severe COVID-19 pneumonia with hyperinflammatory syndrome and acute respiratory failure: A single center study of 100 patients in Brescia, Italy. Autoimmun Rev.

[11] Potere N, Di Nisio M, Cibelli D, Scurti R, Frattari A, Porreca E (2021). Interleukin-6 receptor blockade with subcutaneous tocilizumab in severe COVID-19 pneumonia and hyperinflammation: a case-control study. Ann Rheum Dis.

[12] Eimer J, Vesterbacka J, Svensson AK, Stojanovic B, Wagrell C, Sonnerborg A (2021). Tocilizumab shortens time on mechanical ventilation and length of hospital stay in patients with severe COVID-19: a retrospective cohort study. J Intern Med.

[13] Colaneri M, Bogliolo L, Valsecchi P, Sacchi P, Zuccaro V, Brandolino F (2020). Tocilizumab for Treatment of Severe COVID-19 Patients: Preliminary Results from SMAtteo COvid19 REgistry (SMACORE). Microorganisms.

[14] Hermine O, Mariette X, Tharaux PL, Resche-Rigon M, Porcher R, Ravaud P (2021). Effect of Tocilizumab vs Usual Care in Adults Hospitalized With COVID-19 and Moderate or Severe Pneumonia: A Randomized Clinical Trial. Jama Intern Med.

[15] Stone JH, Frigault MJ, Serling-Boyd NJ, Fernandes AD, Harvey L, Foulkes AS (2020). Efficacy of Tocilizumab in Patients Hospitalized with Covid-19. N Engl J Med.

[16] Kotak S, Khatri M, Malik M, Malik M, Hassan W, Amjad A (2020). Use of Tocilizumab in COVID-19: A Systematic Review and Meta-Analysis of Current Evidence. Cureus.

[17] the National Health Commission of China, Diagnosis and Treatment Protocol for Novel Coronavirus Infection-Induced Pneumonia (Version 7).

[18] Chousterman BG, Swirski FK, Weber GF (2017). Cytokine storm and sepsis disease pathogenesis. Semin Immunopathol.

[19] Chen X, Zhao B, Qu Y, Chen Y, Xiong J, Feng Y (2020). Detectable Serum Severe Acute Respiratory Syndrome Coronavirus 2 Viral Load (RNAemia) Is Closely Correlated With Drastically Elevated Interleukin 6 Level in Critically Ill Patients With Coronavirus Disease 2019. Clin Infect Dis.

[20] Vasileva D, Badawi A (2019). C-reactive protein as a biomarker of severe H1N1 influenza. Inflamm Res.

[21] Wang D, Hu B, Hu C, Zhu F, Liu X, Zhang J (2020). Clinical Characteristics of 138 Hospitalized Patients With 2019 Novel Coronavirus-Infected Pneumonia in Wuhan, China. JAMA.

[22] Xu Z, Shi L, Wang Y, Zhang J, Huang L, Zhang C (2020). Pathological findings of COVID-19 associated with acute respiratory distress syndrome. Lancet Respir Med.

[23] Tanaka T, Narazaki M, Kishimoto T (2014). IL-6 in inflammation, immunity, and disease. Cold Spring Harb Perspect Biol.

[24] Mehta P, McAuley DF, Brown M, Sanchez E, Tattersall RS, Manson JJ (2020). COVID-19: consider cytokine storm syndromes and immunosuppression. Lancet.

[25] Martinez-Sanz J, Muriel A, Ron R, Herrera S, Perez-Molina JA, Moreno S (2021). Effects of tocilizumab on mortality in hospitalized patients with COVID-19: a multicentre cohort study. Clin Microbiol Infect.

[26] Luo P, Liu Y, Qiu L, Liu X, Liu D, Li J (2020). Tocilizumab treatment in COVID-19: A single center experience. J Med Virol.

[27] Kow CS, Hasan SS (2021). The effect of tocilizumab on mortality in hospitalized patients with COVID-19: a meta-analysis of randomized controlled trials. Eur J Clin Pharmacol.

[28] Hariyanto TI, Hardyson W, Kurniawan A (2021). Efficacy and Safety of Tocilizumab for Coronavirus Disease 2019 (Covid-19) Patients: A Systematic Review and Meta-analysis. Drug Res (Stuttg).

[29] Zhao M, Lu J, Tang Y, Dai Y, Zhou J, Wu Y (2021). Tocilizumab for treating COVID-19: a systemic review and meta-analysis of retrospective studies. Eur J Clin Pharmacol.

[30] Capra R, De Rossi N, Mattioli F, Romanelli G, Scarpazza C, Sormani MP (2020). Impact of low dose tocilizumab on mortality rate in patients with COVID-19 related pneumonia. Eur J Intern Med.

