# Supplementary material for: Inflammation characteristics and anti-inflammation treatment with tocilizumab of severe/critical COVID-19 patients: A retrospective cohort study
Source: Int J Biol Sci. 2021 May 17;17(8):2124–34. doi: 10.7150/ijbs.56952 (PMC8193270; doi:10.7150/ijbs.56952)
Supplement: Supplementary file 1 — Supplementary figures and tables. [file ijbsv17p2124s1.pdf]

Supplementary materials

Supplementary Table 1 Laboratory findings of severe/critical patients with COVID-19

Supplementary Table 2 Multivariable Cox regression analysis of risk factors associated with in-hospital death of severe/critical patients with COVID-19

Supplemental Table 3 Characteristics between the tocilizumab and non-tocilizumab group in severe/critical COVID-19 patients before and after matching

Supplementary Table 4 Effects of tocilizumab treatment on inflammation-related laboratory indicators

Supplementary Figure 1 Survival curves by tocilizumab use, with propensity matching in severe/critical patients with COVID-19

Supplementary Table 1 Laboratory findings of severe/critical patients with COVID-19

Parameters	Total (n=727)	Deaths (n=42)	Recovered patients (n=685)	P value
**Laboratory findings (normal range)**				
White blood cell count, $\times 10^9$/L (3.5-9.5)				
First test	6.00 (4.80-7.80)	8.60 (6.60-13.70)	6.00 (4.80-7.50)	<0.001
Last test	5.90 (4.90-7.53)	12.30 (7.43-21.38)	5.80 (4.90-7.20)	<0.001
Neutrophil ratio, % (40-75)				
First test	65.65 (57.33-76.20)	88.20 (78.00-92.50)	64.90 (57.10-74.45)	<0.001
Last test	61.90 (55.00-70.40)	93.00 (88.15-95.68)	60.65 (54.73-68.78)	<0.001
Lymphocyte ratio, % (20-50)				
First test	23.00 (14.30-30.20)	6.30 (4.20-10.80)	23.70 (15.63-30.68)	<0.001
Last test	26.90 (18.30-32.70)	3.65 (2.65-7.53)	27.35 (20.40-32.98)	<0.001
Monocyte ratio, % (3-10)				
First test	7.40 (5.90-9.00)	4.40 (2.10-6.30)	7.50 (6.10-9.10)	<0.001
Last test	7.80 (6.40-9.28)	2.05 (1.20-4.18)	7.90 (6.70-9.30)	<0.001
Neutrophil count, $\times 10^9$/L (1.8-6.3)				
First test	3.89 (2.83-5.53)	7.53 (5.56-12.48)	3.79 (2.79-5.24)	<0.001
Last test	3.68 (2.77-4.90)	11.45 (6.67-19.85)	3.60 (2.74-4.65)	<0.001
Lymphocyte count, $\times 10^9$/L (1.1-3.2)				
First test	1.32 (0.89-1.71)	0.57 (0.43-1.03)	1.37 (0.94-1.73)	<0.001
Last test	1.49 (1.04-1.89)	0.42 (0.31-0.83)	1.54 (1.10-1.93)	<0.001
Monocyte count, $\times 10^9$/L (0.1-0.6)				
First test	0.46 (0.36-0.58)	0.37 (0.21-0.62)	0.44 (0.34-0.57)	0.082
Last test	0.46 (0.36-0.58)	0.31 (0.13-0.53)	0.47 (0.37-0.58)	0.002
Neutrophil/Lymphocyte ratio				
First test	2.76 (1.85-5.10)	13.21 (7.88-17.83)	2.71 (1.83-4.62)	<0.001
Last test	2.41 (1.69-4.23)	16.05 (8.97-33.16)	2.33 (1.66-3.86)	<0.001
Interleukin-6, pg/mL (0-7)				
First test	2.74 (1.50-8.99)	83.73 (39.39-185.28)	2.66 (1.50-7.36)	<0.001
last test	4.54 (1.50-37.15)	1848.00 (216.32-5000.00+ [α])	4.07 (1.50-25.00)	<0.001
Procalcitonin, ng/mL (0-0.05)				
First test	0.05 (0.04-0.10)	0.28 (0.12-0.81)	0.05 (0.03-0.09)	<0.001
Last test	0.05 (0.03-0.08)	3.44 (0.66-8.93)	0.05 (0.03-0.07)	<0.001
C-reactive protein, mg/L (0-5)				
First test	4.43 (1.40-22.77)	72.75 (42.67-138.96)	3.85 (1.32-15.90)	<0.001
Last test	2.47 (0.95-8.16)	112.53 (83.59-177.50)	2.12 (0.90-6.05)	<0.001
hs-CRP, mg/L (0-5)				

First test (no. of value > 5 mg/L)	319/727 (43.9%)	31/42 (73.8%)	288/685 (42.0%)	<0.001
Last test (no. of value > 5 mg/L)	143/727 (19.7%)	25/42 (59.5%)	118/685 (17.2%)	<0.001

Data are median (IQR), or n (%), or n/N (%). First test means the first-tested value after admission and last test means the last-tested value after admission. P values were calculated by Mann-Whitney U test, χ^2 test, or Fisher's exact test, as appropriate.

+ $^{\alpha}$ means that the value exceeded the upper limit of detection.

IQR=interquartile range; hs-CRP=High sensitivity C-reactive protein

Supplementary Table 2 Multivariable Cox regression analysis of risk factors associated with in-hospital death of severe/critical patients with COVID-19

	**Adjusted HR[a] (95% CI)**	**P value**
**Clinical Characteristics**		
Age, years	1.026 (0.996-1.056)	0.085
Female Sex (vs Male)	0.723 (0.342-1.528)	0.395
Classification of disease at admission (critical vs severe)	5.996 (2.667-13.481)	<0.001
Comorbidity		
Coronary heart disease	3.645 (1.511-8.795)	0.004
Diabetes	1.272 (0.551-2.938)	0.573
Other CVDs	0.542 (0.187-1.567)	0.258
Respiratory diseases	1.965 (0.897-4.306)	0.092
Chronic renal diseases	1.193 (0.400-3.562)	0.752
Other cancers	0.343 (0.074-1.579)	0.169
**Therapies**		
Antibiotics therapy	4.387 (1.464-13.149)	0.008
Blood purification	1.983 (0.346-11.357)	0.442
Hemodialysis	0.469 (0.075-2.940)	0.418
Convalescent plasma	2.352 (1.093-5.061)	0.029
Corticosteroids	8.727 (3.859-19.734)	<0.001
Traditional Chinese medicine	0.072 (0.035-0.149)	<0.001
Tocilizumab	0.103 (0.013-0.798)	0.033

HR=hazard ratio; CI= confidence interval；CVDs=cardiovascular diseases

[a]Multivariable Cox regression analysis was performed by adjusting age, gender, classification of disease severity, comorbiditis, antibiotics, convalescent plasma, corticosteroids, blood purification, hemodialysis and traditional Chinese medicine.

Supplemental Table 3 Characteristics between the tocilizumab and non-tocilizumab group in severe/critical COVID-19 patients before and after matching

Variables	Before matching			After matching		
	Non-tocilizumab group	Tocilizumab group	P value	Non-tocilizumab group	Tocilizumab group	P value
**Number of cases**	677	50		50	50	
**Clinical characteristics**						
Age, years	64.0 (56.0-72.0)	71.0 (64.7-77.2)	<0.001	69.0 (62.7-76.2)	71.0 (64.7-77.2)	0.238
Sex			0.133			1.000
Male	359 (53.0%)	32 (64.0%)		32 (64.0%)	32 (64.0%)	
Female	318 (47.0%)	18 (36.0%)		18 (36.0%)	18 (36.0%)	
Classification of disease severity at admission			0.892			1.000
Severe	643 (95.0%)	47 (94.0%)		47 (94.0%)	47 (94.0%)	
Critical	34 (5.0%)	3 (6.0%)		3 (6.0%)	3 (6.0%)	
Current Smoker	48 (7.1%)	4 (8.0%)	1.000	5 (10.0%)	4 (8.0%)	1.000
Comorbidity	430 (63.5%)	39 (78.0%)	0.039	40 (80.0%)	39 (78.0%)	1.000
Hypertension	293 (43.3%)	26 (52.0%)	0.230	28 (56.0%)	26 (52.0%)	0.832
Coronary heart disease	72 (10.6%)	8 (16.0%)	0.242	7 (14.0%)	8 (16.0%)	1.000
Diabetes	136 (20.1%)	10 (20.0%)	0.988	11 (22.0%)	10 (20.0%)	1.000
Other cardiovascular diseases	75 (11.1%)	7 (14.0%)	0.529	4 (8.0%)	7 (14.0%)	0.549
COPD	12 (1.8%)	1 (2.0%)	0.607	4 (8.0%)	1 (2.0%)	0.375
Other chronic lung illness	32 (4.7%)	3 (6.0%)	0.727	5 (10.0%)	3 (6.0%)	0.727
Other diseases	99 (14.6%)	6 (12.0%)	0.611	5 (10.0%)	6 (12.0%)	1.000
**Treatments**						
Nasal catheter oxygen	536 (79.2%)	41 (82.0%)	0.634	45 (90.0%)	41 (82.0%)	0.344
High flow nasal cannula oxygen therapy	63 (9.3%)	15 (30.0%)	0.000	11 (22.0%)	15 (30.0%)	0.481
Non-Invasive Mechanical Ventlilation	45 (6.6%)	9 (18.0%)	0.007	7 (14.0%)	9 (18.0%)	0.774
Invasive Mechanical Ventlilation	27 (4.0%)	5 (10.0%)	0.100	4 (8.0%)	5 (10.0%)	1.000
Loop support	19 (2.8%)	3 (6.0%)	0.398	4 (8.0%)	3 (6.0%)	1.000
Blood purification	6 (0.9%)	0 (0.0%)	1.000	1 (2.0%)	0 (0.0%)	1.000
Antibiotics	284 (41.9%)	31 (56.0%)	0.006	26 (52.0%)	31 (56.0%)	0.405
Antivirals	394 (58.2%)	28 (70.0%)	0.761	31 (62.0%)	28 (70.0%)	0.678
Corticosteroids	160 (23.6%)	25 (50.0%)	0.000	24 (46.0%)	25 (50.0%)	0.832
Traditional Chinese medicine	584 (86.3%)	43 (86.0%)	0.455	48 (96.0%)	43 (86.0%)	0.508
Convalescent plasma	64 (9.5%)	17 (34.0%)	0.000	10 (20.0%)	17 (34.0%)	0.092

Intravenous albumin	112 (16.5%)	21 (42.0%)	0.000	19 (38.0%)	21 (42.0%)	0.824

Values are presented as median (IQR) or number; P values were compared by Chi-square test, Fisher's exact test, Mann-Whitney U test, McNemar test, or Wilcoxon signed ranks test.
COPD=chronic obstructive pulmonary disease; IQR=interquartile range

Supplementary Table 4 Effects of tocilizumab treatment on inflammation-related laboratory indicators

Parameters	Before tocilizumab treatment	day 3 after tocilizumab treatment	day 7 after tocilizumab treatment	day 14 after tocilizumab treatment
Interleukin-6, pg/mL	17.91 (10.48-30.80)	101.10 (63.70-343.00)	63.55 (45.84-217.63)	31.08 (8.24-59.71)
Procalcitonin, ng/mL	0.09 (0.07-0.21)	0.08 (0.03-0.26)	0.05 (0.03-0.07)	0.05 (0.04-0.08)
C-reactive protein, mg/L	12.52 (5.92-37.50)	4.34 (1.57-15.47)	1.49 (0.31-3.31)	0.42 (0.13-2.35)
hs-CRP (no. of value >5 mg/L)	40/49 (81.63%)	19/43 (44.18%)	5/44 (11.36%)	4/36 (11.11%)
Neutrophil ratio, %	68.90 (64.07-78.77)	57.95 (51.37-65.82)	54.95 (49.75-64.40)	58.70 (46.55-62.07)
Neutrophil count, $\times 10^9$/L	4.45 (3.18-6.71)	2.59 (1.83-3.62)	2.79 (1.87-4.16)	3.03 (1.87-4.05)
Lymphocyte ratio, %	19.85 (11.42-23.80)	28.05 (18.92-34.17)	29.85 (20.77-34.60)	30.20 (27.22-39.40)
Lymphocyte count, $\times 10^9$/L	1.06 (0.73-1.42)	1.05 (0.73-1.55)	1.30 (1.12-1.76)	1.61 (1.17-2.42)

Data are median (IQR), or n/N (%).

hs-CRP=High sensitivity C-reactive protein; IQR=interquartile range

Supplementary Figure 1 Survival curves by tocilizumab use, with propensity matching in severe/critical patients with COVID-19

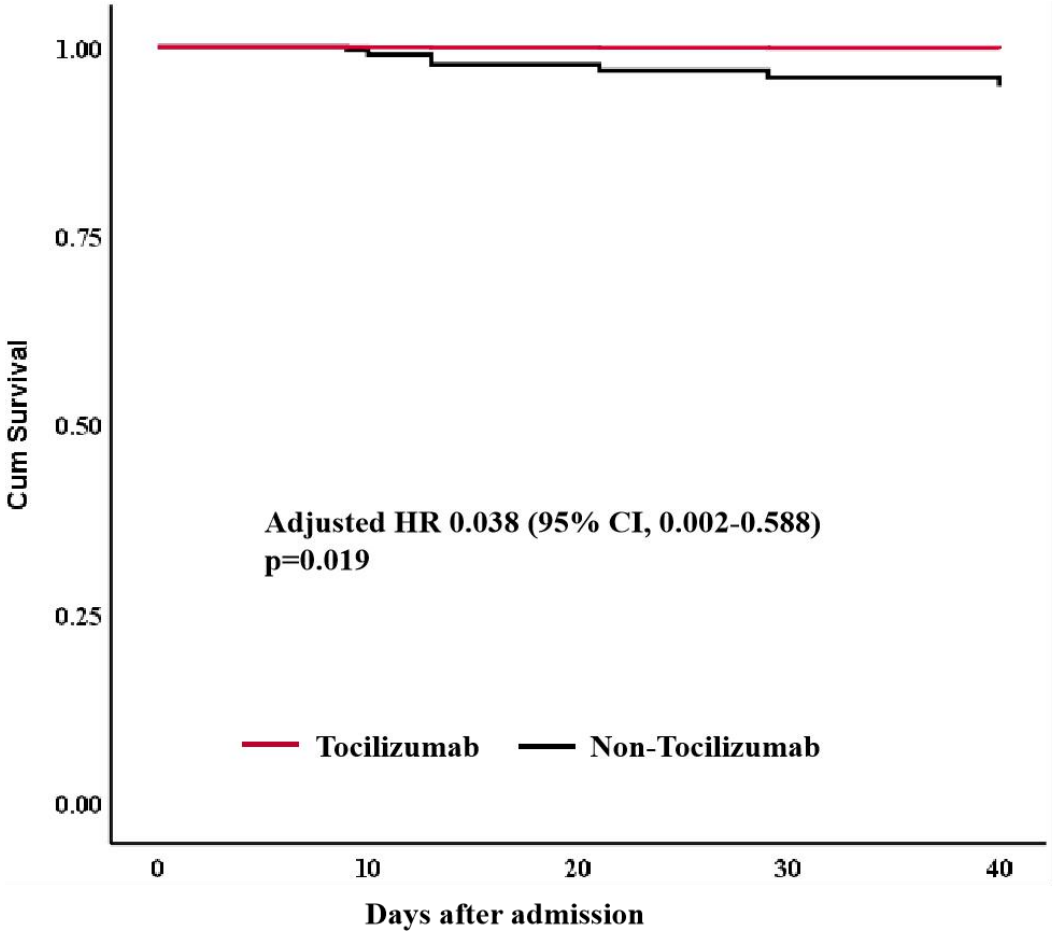

Adjusted HR and P value were calculated using Cox regression model with adjustment of age, coronary disease, the use of corticosteroids and the use of traditional Chinese medicine. In this panel, the red line represents tocilizumab use and the black line represents no tocilizumab use. The Y axis is cumulative survival, the X axis is hospital days. Matching caliper 0.05. HR = Hazard Ratio; CI = Confidence Intervals.

Supplementary Materials

Author information:

Qin Hu[1, #], Yu Xu[2, #], Ying Xiang[1, #], Bin Wang[2], Zhiquan Yuan[1], Yifan Shan[1], Wenjing Zhang[2], Na Wu[1], Tingting Xia, Chengying Li[1], Weijia Xie[1], Xiaoyue Jia[1], Zubin Yu[3, *], Li Bai[2, *], Yafei Li[1, *]

[1] *Department of Epidemiology, College of Preventive Medicine, Army Medical University (Third Military Medical University), Chongqing 400038, People's Republic of China*

[2] *Department of Respiratory and Critical Care Medicine, the Second Affiliated Hospital of Army Medical University, Chongqing 400037, People's Republic of China*

[3] *Department of Thoracic Surgery, the Second Affiliated Hospital of Army Medical University, Chongqing 400037, People's Republic of China*

Correspondence:

Yafei Li, Ph.D.

Department of Epidemiology, College of Preventive Medicine, Army Medical University (Third Military Medical University), NO.30 Gaotanyan Street, Chongqing 400038, People's Republic of China

E-mail: liyafei2008@tmmu.edu.cn; liyafei2008@hotmail.com

Telephone: +86 23 68752293

E-mail of authors:

Qin Hu: qin_hu1003@hotmail.com

Yu Xu: cqxuyu1982@tmmu.edu.cn

Ying Xiang: 83127624@qq.com

Bin Wang: wangbin985@126.com

Zhiquan Yuan: 498625450@qq.com

Yifan Shan: shan1fan@163.com

Wenjing Zhang: 375303335@qq.com

Na Wu: cqwuna@126.com

Tingting Xia: 451160753@qq.com

Chengying Li: 15025334162@163.com

Weijia xie: xieweijia33@hotmail.com

Xiaoyue Jia: 675465936@qq.com

Zubin Yu：yzbpost@126.com

Li Bai: blpost@126.com
